# Improvement of culture conditions for long-term in vitro culture of *Plasmodium vivax*

**DOI:** 10.1186/s12936-015-0815-z

**Published:** 2015-08-05

**Authors:** Wanlapa Roobsoong, Chayada S Tharinjaroen, Nattawan Rachaphaew, Porpimon Chobson, Louis Schofield, Liwang Cui, John H Adams, Jetsumon Sattabongkot

**Affiliations:** Mahidol Vivax Research Unit, Faculty of Tropical Medicine, Mahidol University, Bangkok, Thailand; Division of Clinical Microbiology, Department of Medical Technology, Faculty of Associated Medical Sciences, Chiang Mai University, Chiang Mai, Thailand; Department of Infectious Diseases, Walter and Elisa Hall Institute of Medical Research, Melbourne, Australia; Department of Entomology, The Pennsylvania State University, University park, PA USA; Department of Global Health, University of South Florida, Tampa, USA

**Keywords:** *Plasmodium vivax*, In vitro culture, Invasion assay, Membrane feeding assay, Culture medium, Reticulocytes

## Abstract

**Background:**

The study of the biology, transmission and pathogenesis of *Plasmodium vivax* is hindered due to the lack of a robustly propagating, continuous culture of this parasite. The current culture system for *P. vivax* parasites still suffered from consistency and difficulties in long-term maintenance of parasites in culture and for providing sufficient biological materials for studying parasite biology. Therefore, further improvement of culture conditions for *P. vivax* is needed.

**Methods:**

Clinical samples were collected from patients diagnosed with *P. vivax* in western Thailand. Leukocyte-depleted *P. vivax* infected blood samples were cultured in a modified McCoy’s 5A medium at 5% haematocrit under hypoxic condition (5% O_2_, 5% CO_2_, and 90% N_2_). Reticulocytes purified from adult peripheral blood were added daily to maintain 4% reticulocytes. Parasites were detected by microscopic examination of Giemsa-stained smears and molecular methods.

**Results:**

The effects of culture variables were first analysed in order to improve the culture conditions for *P. vivax*. Through analysis of the sources of host reticulocytes and nutrients of culture medium, the culture conditions better supporting in vitro growth and maturation of the parasites were identified. Using this system, three of 30 isolates could be maintained in vitro for over 26 months albeit parasite density is low.

**Conclusions:**

Based on the analysis of different culture variables, an improved and feasible protocol for continuous culture of *P. vivax* was developed.

**Electronic supplementary material:**

The online version of this article (doi:10.1186/s12936-015-0815-z) contains supplementary material, which is available to authorized users.

## Background

*Plasmodium vivax* is considered to be the most widely distributed human malaria parasite in tropical and temperate countries [1]. Study of the biology of *P. vivax* is hindered due to the lack of a robustly propagating, continuous culture of this parasite. Several attempts have been made to grow *P. vivax* in vitro; some successfully established conditions for short-term culture [2–4], whereas others improved culture conditions that potentially support longer-term culture of this parasite [5–7]. However, the current lack of a continuous culture system for *P. vivax* requires constant accesses to freshly collected or cryopreserved parasites. Therefore, improvement of in vitro culture methods for *P. vivax* is needed to enhance study of its unique biology, especially underlying mechanisms of transmission and relapse, to develop better tools for elimination [8–10].

In this study, the effects of culture variables were further analysed in order to improve longer term in vitro culture conditions for *P. vivax*. Through empiric evaluation of the sources of the host reticulocytes and nutrients of culture medium, the culture conditions better supporting in vitro growth and maturation of the parasites were identified. Based on the optimized conditions, a more simplified and feasible protocol for continuous culture of *P. vivax* was developed.

## Methods

### List of reagents

McCoy’5A (M-4892, Sigma), RPMI 1640 (31800-022, Gibco), Waymouth’s medium (31220023, Gibco), Iscove’s modified Dulbecco’s medium (IMDM) (12440-053, Gibco), orotic acid (02750-10G, Sigma), l-ascobic acid (A4544-100G, Sigma), Inosine 5′-monophosphate disodium salt (I4625-25G), l-glutathione reduced (G6013-100G, Sigma), HT supplement (11067-030, Gibco), cholesterol lipid concentrate (12531-016, Gibco), ACK red blood cell lysis buffer (A10492-01, Gibco), Pen/Strep solution (15140-122, Gibco), erythropoietin (EPO) (Exprex4000, Janssen-Cilag, Australia), Stem cell factor (SCF) (produced in-house at WEHI), Histopaque (10771, Sigma), Nycoprep Universal 60% solution (AN1106865, Axis-Shield).

### Collection of *Plasmodium vivax*-infected blood

Clinical isolates of *P. vivax* were collected from symptomatic patients attending four malaria clinics in Saiyok Distict, Kanchachaburi Province, Thailand. The protocol was approved by the Ethical Review Committee of the Faculty of Tropical Medicine, Mahidol University (MUTM 2011-040-03). After informed consent was obtained, 20 mL of blood were drawn into a 50 mL tube containing lithium-heparin. Infected blood was washed once with RPMI 1640. Leukocytes were removed by passing the infected blood at 50% haematocrit through pre-equilibrated Plasmodipur^®^ filter (Europroxima). Thick and thin blood smears were prepared from 1 µL of packed cells before and after passing through Plasmodipur filter. After drying, thin smears were fixed with methanol and stained with 10% Giemsa for 10 min. Parasite density was determined from Giemsa-stained thick smear.

### Reticulocyte preparation

Peripheral blood (type O) from healthy donors collected in CPDA1 was obtained from the Blood Bank Unit. Cord blood samples were obtained from the Rajavithi Hospital in Bangkok. The protocol for cord blood collection was approved by the Ethical Review Committee of the Rajavithi Hospital (MUTM2011-030-01). Leukocytes were removed by passing the whole blood through PALL^®^ leukocyte reduction filter (RC2VAE, Haemonetics Corporation). Leukocyte-depleted blood was centrifuged at 1,000×*g* for 10 min to remove plasma. Packed blood cells were washed twice with RPMI 1640 incomplete medium (RIM) (RPMI 1640, HEPES 5.96 g/L, NaHCO_3_ 2.0 g/L, d-glucose 1.0 g/L and gentamycin 40 mg/mL) and centrifuged at 1,000×*g* for 10 min then resuspended to 20% haematocrit in RIM. Nycodenz was diluted to 19% with a KCl buffer (115 mM KCl, 20 mM HEPES, 1 mM MgCl_2_, 1 mM NaH_2_PO_4_, 10 mM d-glucose, 0.5 mM EGTA, and 12 mM NaCl, pH 7.4). Diluted blood was overlaid on 19% Nycodenz and centrifuged at 3,000×*g* for 30 min. Reticulocyte-enriched fraction was collected from the interface and washed three times with RIM by centrifugation at 1,000×*g* for 10 min. Enriched reticulocytes were resuspended to 50% haematocrit with RIM. Enriched reticulocytes were kept at 4°C for a maximum of 2 weeks from the date of blood collection to avoid the loss of erythrocyte surface markers. Enriched reticulocytes were stained with New Methylene Blue (Retic chex) for 15 min before making thin smears. The percentage of reticulocytes was determined by counting the number of reticulin-containing cells from 5,000 cells. Cells with two or more dots of reticulin were considered reticulocytes.

### Isolation of CD34^+^ stem cells

Packed cells of buffy coat from adult donors were obtained from the Australian Red Cross Blood Services (HREC 13/04) and used for the isolation of CD34^+^ stem cells. The packed cells were diluted with an equal volume of Hank’s Balanced Salt Solution/Acid Citrate Dextrose (HBSS/ACD), overlaid on 20 mL of Histopaque, and centrifuged at 400×*g* for 30 min. The purified stem cell fraction was collected and washed three times with RIM. After treatment with 5 mL of red blood cell lysis buffer for 12 min at room temperature, CD34^+^ containing white blood cell fraction was collected. CD34^+^ cells were isolated by using the MiniMacs Direct CD34^+^ Progenitor Cell Isolation kit (Miltenyi Biotech 130-046-703) according to the manufacturer’s instructions. Approximately 5 × 10^8^ mononuclear cells with a yield of up to 5 × 10^5^ CD34^+^ cells could be obtained from one Buffy pack (50–60 mL).

### Haematopoietic stem cell culture system

The haematopoietic stem cell culture system was composed of three phases: (1) commitment to the erythroid lineage phase, (2) proliferation phase, and (3) differentiation and enucleating phase. The purified CD34^+^ cells were cultured in IMDM culture medium containing 10 µg/mL insulin, 5% human AB serum, and 1% Pen/Strep at a concentration of 0.5–2 × 10^6^ cells/mL for 18–19 days with daily change of the culture medium. The first phase medium contained IL-3 (1 ng/mL), SCF (40 ng/mL), transferrin (200 µg/mL) and EPO (3 U/mL). The second phase medium contained SCF, EPO and transferrin (1,000 µg/mL). The third phase medium contained EPO and transferrin (1,000 µg/mL). After 19 days of culture, the original cell population has amplified several thousand times to yield a relatively pure reticulocyte population. In order to obtain pure and enucleated reticulocytes, the culture was filtered using leukocyte reduction filters (WBF3, Haemonetics Corporation) to remove the remaining nucleated cells and the nucleus debris. After purification, reticulocytes were stored in saline-anenine-glucose-mannitol for up to 14 days at 4°C.

### Culture medium

Three media were used in this study: McCoy’s 5A medium (McCoy’s 5A, HEPES 5.96 g/L, NaHCO_3_ 2.0 g/L, d-glucose 2.0 g/L, gentamycin 40 mg/mL), RPMI 1640 (RPMI 1640, HEPES 5.96 g/L, NaHCO_3_ 2.0 g/L, d-glucose 1.0 g/L, MgSO_4_ 0.016 g/L, KH_2_PO_4_ 0.026 g/L, CaCl_2_ 0.03 g/L, l-ascorbic acid 0.006 g/L, thiamine 0.010 g/L, hypoxanthine 0.01 g/L, gentamycin 40 mg/mL), and Waymouth’s. Only human AB serum from Duffy positive blood donors were used in all experiments. The human AB serum was heat inactivated at 56°C for 30 min and filtered sterile. To prepare the complete culture medium, each medium was supplemented with either 25 or 50% heat inactivated human AB serum. To prepare Modified McCoy’s 5A medium (MMM), McCoy’s 5A medium was supplemented with 25% heat inactivated human AB serum, hypoxanthine (360 µM), cholesterol (750 mg/L) and a 10 X additives was freshly added to the culture medium. The 10 X additives were prepared by diluting all additives (ascorbic acid 0.0116 g/L, reduced GSH 2.2 mM, glucose 4 g/L, orotic acid 360 µM, inosine 5′-monophosphate 360 µM) in McCoy’s 5A complete medium. MMM was always freshly prepared.

### Long-term in vitro culture of *Plasmodium vivax*

Leukocyte-depleted *P. vivax*-infected blood was cultured in 24-well plates with MMM complete medium at 5% haematocrit (500 µL culture volume). Parasite culture was incubated at 37°C in a hypoxic environment (5% O_2_, 5% CO_2_ and 90% N_2_). Culture medium was changed daily. Reticulocytes were successively added to the culture in order to maintain 4% reticulocytes. Giemsa-stained thick and thin blood smears were prepared from 1 µL packed cells to monitor parasites growth.

### In vitro invasion assay

Leukocyte-depleted *P. vivax*-infected blood was incubated with McCoy’s 5A medium supplemented with 25% heat inactivated human AB serum (standard McCoy’s 5A complete medium) in T75 cm^2^ tissue culture flask. Parasites were cultured as described above until reaching the schizont stage (~18–24 h). Parasite culture was washed once with RIM and centrifuged at 1,000×*g* for 10 min. Packed infected blood was diluted to 20% haematocrit with RIM and overlaid on 45% Percoll (diluted in 1 × PBS, pH 7.4). The schizont-enriched fraction was collected after centrifugation at 1,200×*g* for 20 min from the interface and washed three times with RIM by centrifugation at 500×*g* for 10 min. To optimize the reticulocyte content in the culture, enriched schizonts were incubated with 1, 4, or 8% of reticulocytes obtained from cord blood at a final parasitaemia of 0.01%. The schizont-reticulocyte mixture was cultured in standard McCoy’s 5A complete medium in 24-well plates (5% haematocrit; 500 µL culture volume) and incubated at 37°C under hypoxic conditions. After 18 h, ring-stage parasites were observed microscopically after Giemsa staining. The proportion of ring-stage parasites was determined from 5,000 cells.

### Mosquito membrane feeding assay

Membrane feeding assay was performed according to a published protocol [11]. Parasite culture was transferred to 1.5 mL tubes and spun at 1,000×*g* for 3 min. The culture supernatant was removed and the packed infected blood was washed once with warmed RIM. The packed infected blood was resuspended to 50% haematocrit with warmed human AB serum and offered to 50 female *Anophelesdirus* mosquitoes for 30 min. Engorged mosquitoes were collected and maintained on 10% sugar. Seven days after membrane feeding, mosquitoes were dissected to check the presence of oocysts on the midguts.

### Nested-PCR

Nested-PCR was performed to confirm the presence of *P. vivax* parasites in cultures. Genomic DNA was extracted from 100 µL packed cells with QIAamp^®^ DNA Mini Kit (QIAGEN) according to the manufacture’s protocol. Three primers were used in nested-PCR: P1F forward primer 5′-ACGATCAGATACCGTCGTAATCTT-3′, P2R reverse primer 5′-GAACCCAAAGACTTTGATTTCTCAT-3′, and VR reverse primer 5′-CAATCTAAGAATAAACTCCGAGAGGAAA-3′. The 18s rDNA was first amplified by using P1F and P2R primers and *P. vivax* was confirmed subsequently by using vivax-specific P1F and VR primers.

### RT-QMAL

The RT-QMAL was performed by using published protocol [12]. Fifty microlitres of packed cells from culture was mixed with 250 µL of RNA protect^®^. The total RNA was extracted by using the RNeasy^®^ plus 96 (74192, Qiagen) according to manufacturer’s protocol. To get rid of DNA, the samples were treated with RNase-free DNase Set (79254, Qiagen). To amplify the *P. vivax*-specific 18s rRNA, QMAL forward primer (Qmal_Fw) 5′-TTA GAT TGC TTC CTT CAG TRC CTT ATG-3′, Qmal reverse primer (Qmal_Rev) 5′-TGT TGA GTC AAA TTA AGC CGC AA-3′, Qmal probe 5′-FAM-TCA ATT CTT TTA ACT TTC TCG CTT GCG CGA-BHQ1-3′ and TaqMan^®^ Gene Expression Master mix (4369016, Lifetechnologies) were used. Standard curve was generated from assay-specific control plasmid at concentration of 10^2^–10^6^ copies/reaction. The amplification was performed for 45 cycles on CFX96™ Real-time PCR detection system (Biorad).

## Results

### Optimization of proportion of reticulocytes in culture

**Fig. 1:**
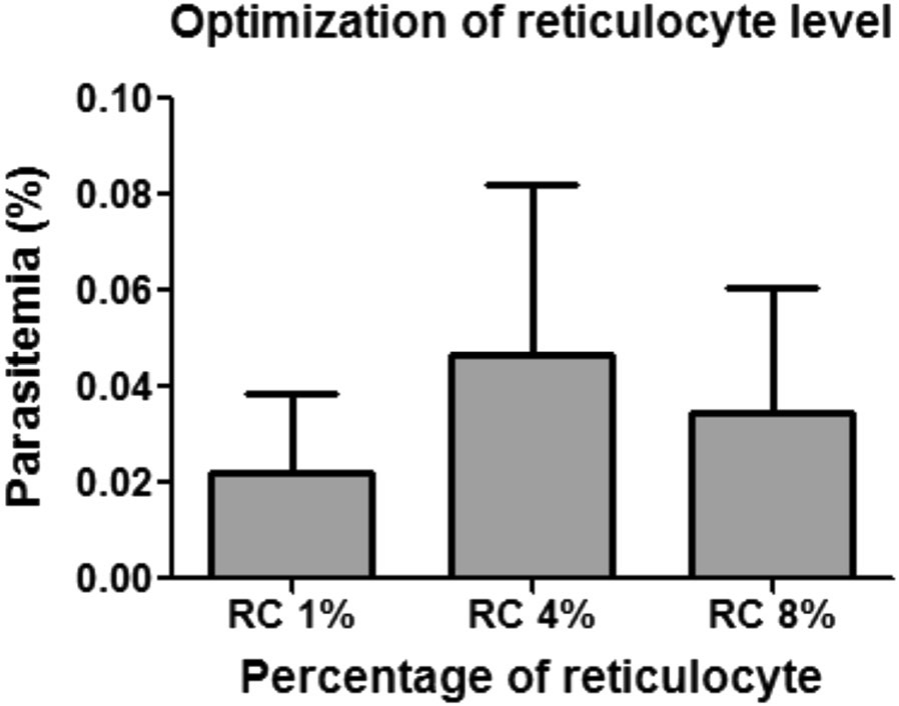
Optimization of reticulocyte levels in culture (N = 9*).* In vitro invasion assay was performed by culturing enriched *P. vivax* schizonts with various percentages of reticulocytes enriched from cord blood (1, 4 and 8%). Ring stage parasitaemia was obtained at 18 h.

Because reticulocytes are required for *P. vivax* invasion, the affect of the proportion of reticulocytes in the in vitro culture was first tested. Using reticulocytes purified from cord blood (RC), the optimization of the proportions of reticulocytes for the invasion of *P. vivax* merozoites was performed with at least nine clinical isolates of *P. vivax*. Purified schizonts (2.5 × 10^4^) were incubated with 1, 4, and 8% (v/v) RC to give a final of parasitaemia of 0.01%. Enumeration of ring-stage parasites 18 h later revealed that with 1% RC the culture contained ~0.02% ring-stage parasitaemia (Fig. 1). An increase of RC to 4% resulted in ~2-fold increase of ring-stage parasitaemia. However, a further increase of RC to 8% did not lead to a significant increase in ring parasitaemia (Fig. 1). Thus a 4% reticulocyte concentration was selected for further optimization of vivax culture conditions.

**Fig. 2:**
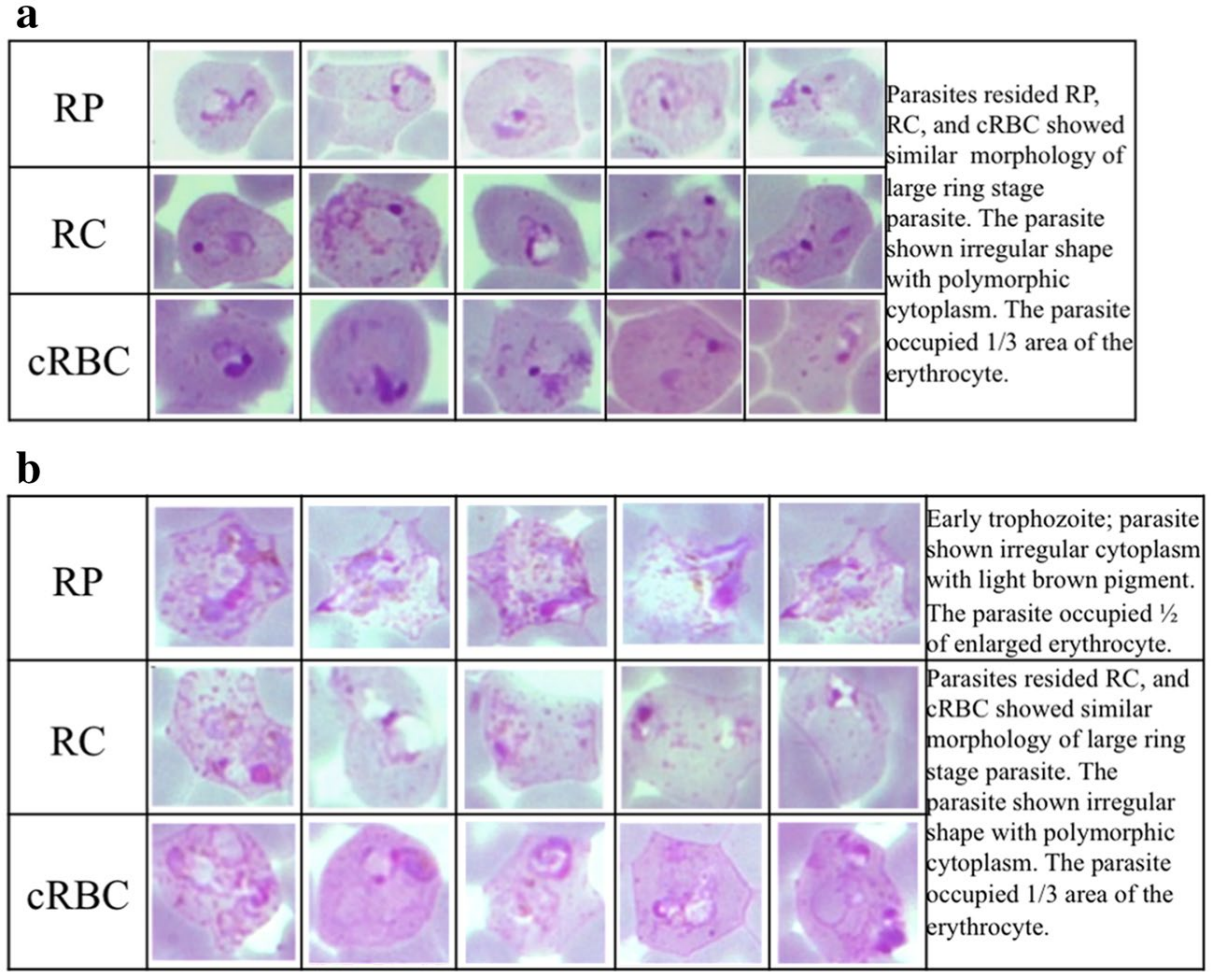
Maturation of *Plasmodium vivax* cultured in different sources of reticulocytes. The invasion assay was performed. The purified schizonts were cultured with 4% of RP, RC, and cRBC to give a final concentration of 0.01% schizonts. The maturation of the parasites was monitored at 18 and 48 h. The developmental assessment of the parasite followed the published guideline [2]. **a** Parasites resided RP, RC, and cRBC at 18 h of cultured showed similar morphology of large ring stage and irregular shape with polymorphic cytoplasm. The parasite occupied 1/3 area of the erythrocyte. **b** The maturation of the parasites were continued to follow at 48 h. Parasites residing RP showed better maturation compared to RC and cRBC. The parasites reside RP developed to early trophozoite with irregular cytoplasm and *light brown* pigment. The parasite occupied ½ of enlarged erythrocyte. While the parasites resided RC, and cRBC showed similar morphology of large ring stage parasite and irregular shape with polymorphic cytoplasm. The parasite occupied 1/3 area of the erythrocyte.

**Fig. 3:**
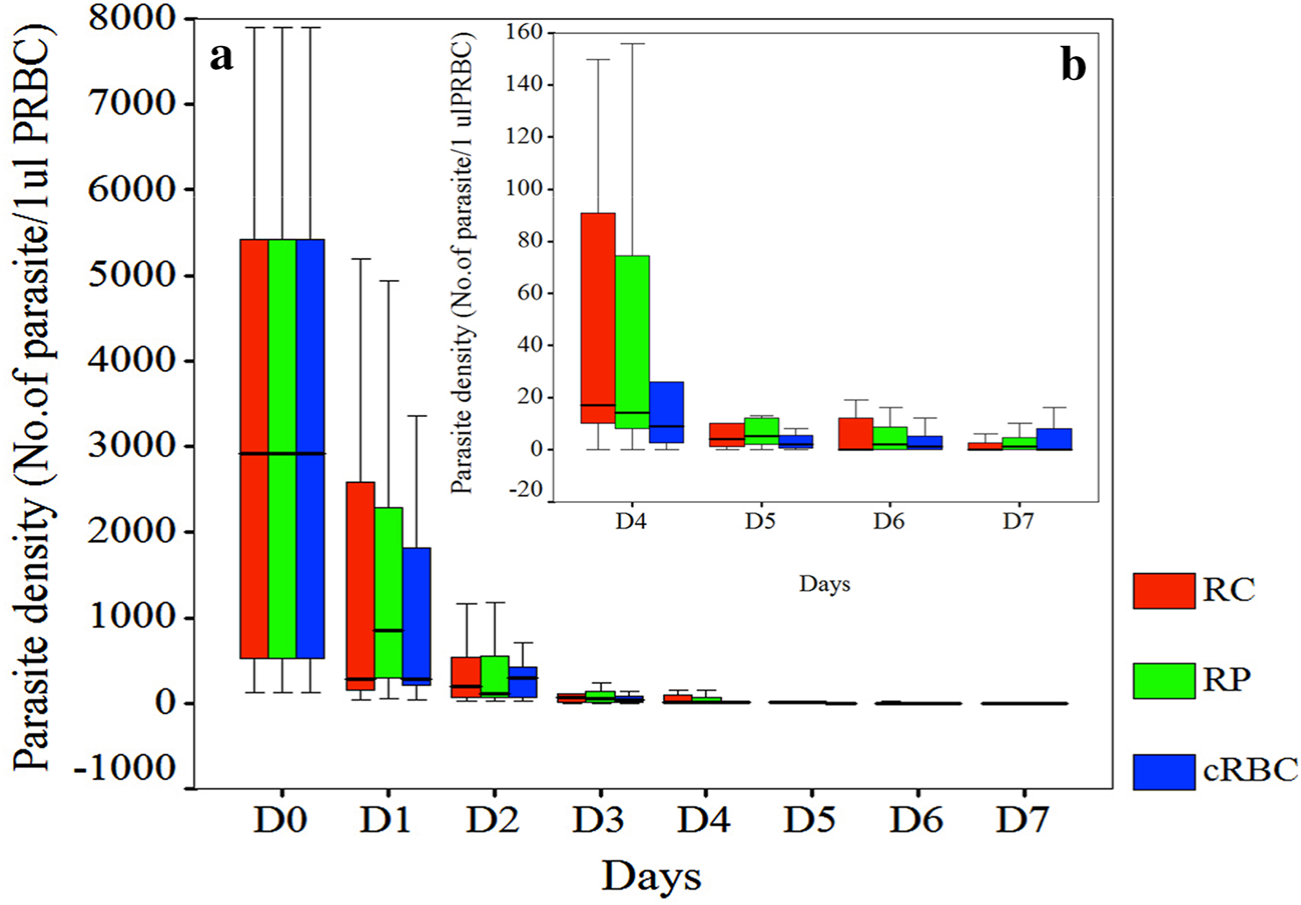
Parasite density of *Plasmodium vivax* cultured with different sources of reticulocytes in 7 days. **a** Parasite density during the 7 day cultured period; **b** Magnification of the graph from day 4 to day 7. Reticulocytes purified from peripheral blood (RP), cord blood (RC) and haematopoietic stem cell (cRBC) were used to cultured fresh isolates of *P. vivax* (10 isolates). Reticulocytes were added to the cultures daily at a final 4% and the parasite density was determined for 7 days from Giemsa-stained thick smears. Each *box* represents average parasite density from 7 days obtained from 10 *P. vivax* isolates.

The influence of sources of reticulocytes to the invasion and maturation of vivax parasites was determined. For comparison, reticulocytes were obtained from two additional sources: purified from peripheral blood (RP), and cultured from haematopoietic stem cells (cRBC). All reticulocytes were seeded at 4% and cultured with the same clinical isolates of *P. vivax.* Invasion and maturation of the parasites in these cultures were monitored at 18 and 48 h, respectively. The published criteria for developmental assessment of *P. vivax* was used to differential stage of the parasite [2]. Ring-stage parasitaemias were not significantly (one way ANOVA, F = 0.192, P = 0.827) different among the three sources of reticulocytes. In addition, the morphology of the parasites residing in RP, RC and cRBC at 18 h did not show obvious differences (Fig. 2a). However, at 48 h parasites in RP showed better maturation than those in RC and cRBC (Fig. 2b). Nonetheless, parasite growth in all three sources of reticulocytes was slightly delayed and none of the parasite isolates tested could complete blood stage schizogony within 48 h. Further, the long-term effect of different sources of reticulocytes on parasite culture was studied with 11 *P. vivax* clinical isolates (Fig. 3). Reticulocytes were added daily to maintain 4% and the parasite density was monitored daily for 7 days. Overall, parasite densities among the three reticulocyte sources were not significantly different (one way ANOVA, F value = 0.024, P = 0.976). Yet, depending on the parasite isolates, some grew better in one source of reticulocytes than others (see Additional file 1). Although RC reticulocytes contain fetal haemoglobin, which was reported to have an inhibitory effect on parasite growth [13], RC better supported parasite maturation as well as gametocyte production in four of the tested parasite isolates (see Additional file 2). Given that RP could support parasite growth for most of the tested isolates, and are a more reliable source of reticulocytes, RP reticulocytes were chosen for subsequent studies to improve long-term culture of *P. vivax*.

### Analysis of culture medium

**Fig. 4:**
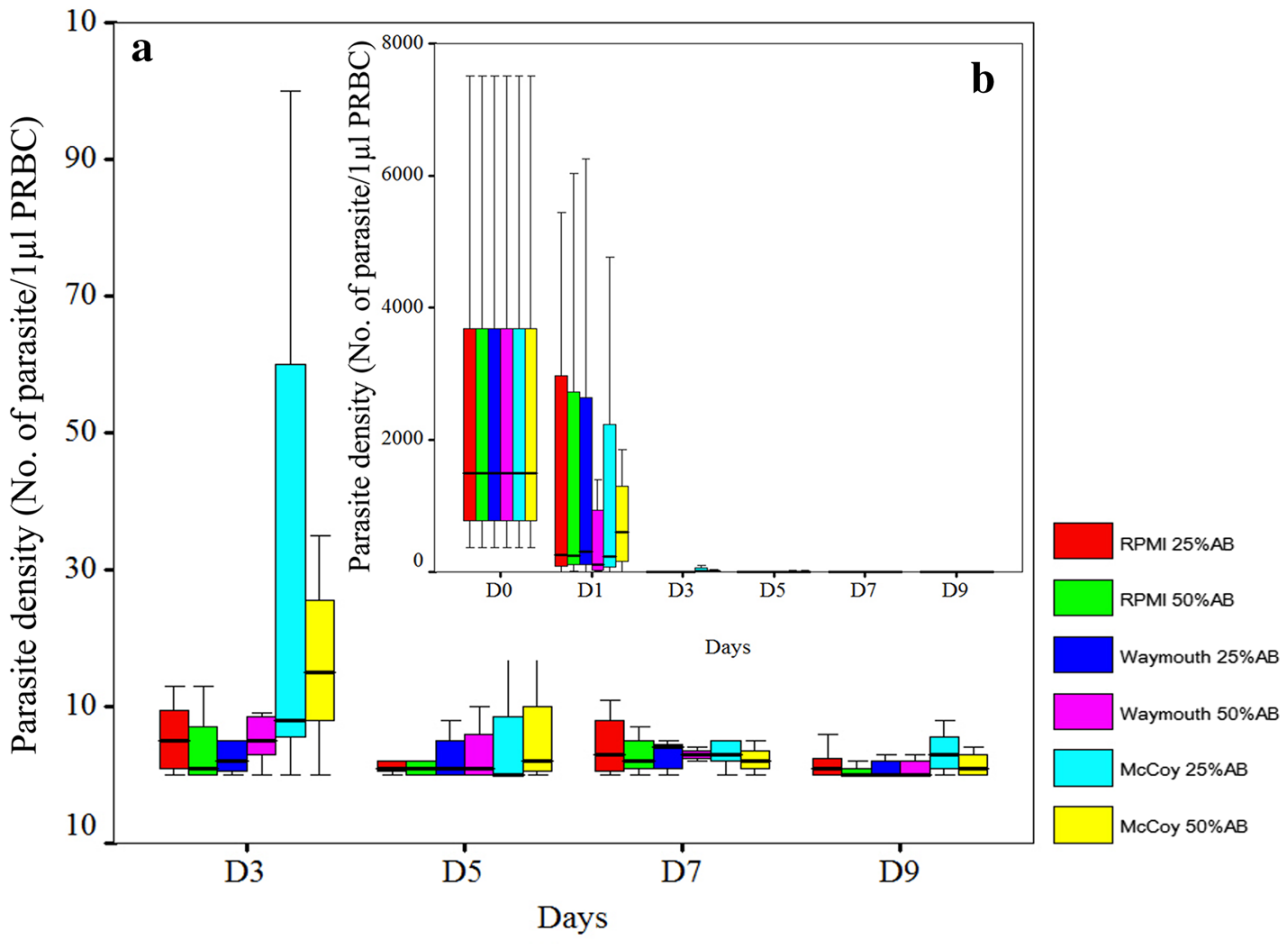
Parasite density of *Plasmodium vivax* cultured with different culture media in 9 days. **a** Parasite density from day 3 to day 9. **b** Parasite density during the 9 day culture period. *P. vivax* parasites (7 isolates) were cultured in McCoy +25%, McCoy +50%, RPMI +25%, RPMI +50%, Waymouth’s +25% and Waymouth’s +50% AB serum. The parasite density was determined for 7 days from Giemsa-stained thick smears. Each *box* represents the parasite density from 7 days obtained from 7 *P. vivax* isolates.

Next we compared three types of culture medium, McCoy’s 5A, RPMI 1640 and Waymouth’s. Human AB serum from only Duffy positive blood donors were used in all experiments. The percentage of serum varied between 25 and 50% for each culture medium, yielding six combinations: McCoy +25% and McCoy +50% serum, RPMI +25% and RPMI +50% serum, and Waymouth’s +25% and Waymouth’s +50% serum. The relative effect of each culture medium-serum combination on parasite growth of seven isolates was determined by counting parasite density in Giemsa-stained thick smears every other day for 9 days (Fig. 4). For RPMI 1640 and Waymouth’s medium, an increase from 25 to 50% serum did not result in discernable differences in parasite densities. However, the effect of serum content in the culture medium was more pronounced for McCoy’s 5A medium. During the first few days of the culture, *P. vivax* parasite cultured in McCoy +50% AB had higher parasite density than in McCoy +25% AB. Yet, during the later days of culture, this trend was reversed with McCoy +25% AB having the higher parasite density. Overall, McCoy +25% showed greater parasite densities and was, therefore, selected as the standard culture medium in this system.

### Modification of the culture medium

**Fig. 5:**
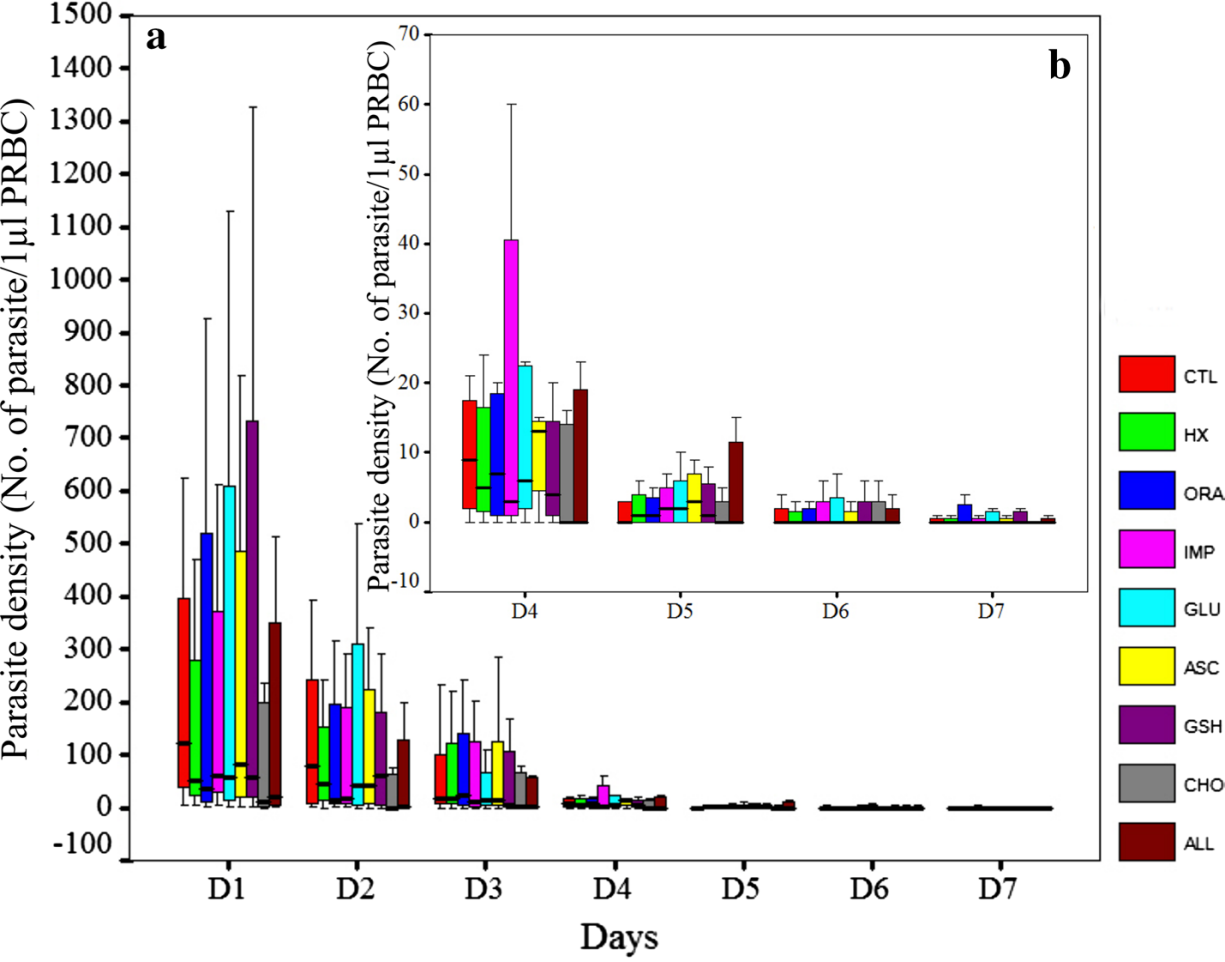
Parasite density of *Plasmodium vivax* cultured with standard McCoy’s 5A medium supplemented with different additives during a 7-day culture period. **a** Parasite density in 7 days. **b** Magnification of the graph from day 4 to day 7. *P. vivax* cultured with standard McCoy’s 5A complete medium (CTL) was used as a control. McCoy’s 5A complete medium was supplemented with each individual additive (CHO, GSH, ASC, Glu, IMP, ORA, HX) or combined all additives (ALL). Data for each *box* represent parasite density of 7 *P. vivax* isolates cultured during 7 days.

**Fig. 6:**
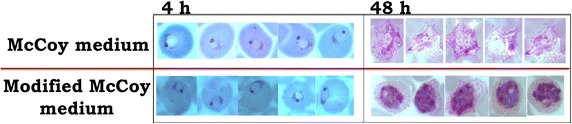
Giemsa staining of *Plasmodium vivax* parasites cultured in McCoy’s 5A medium and MMM. The invasion assay was performed by cultured purified schizonts with RP in 2 different culture media, McCoy’s 5A medium and MMM medium. At 4 h of cultured in both media, parasites successfully reinvaded the new reticulocytes and formed ring stage parasites. When the maturation was continued to observe at 48 h, parasites cultured in MMM medium developed to mature schizont stage which contained more than 5 nuclei and *brown* pigment. The infected erythrocytes were enlarged, very pale staining and contained *pink dots* (Schüffner’s dots) scattered through out the cytoplasm. While the parasites cultured in McCoy’s 5A medium has not reached the schizont stage.

**Fig. 7:**
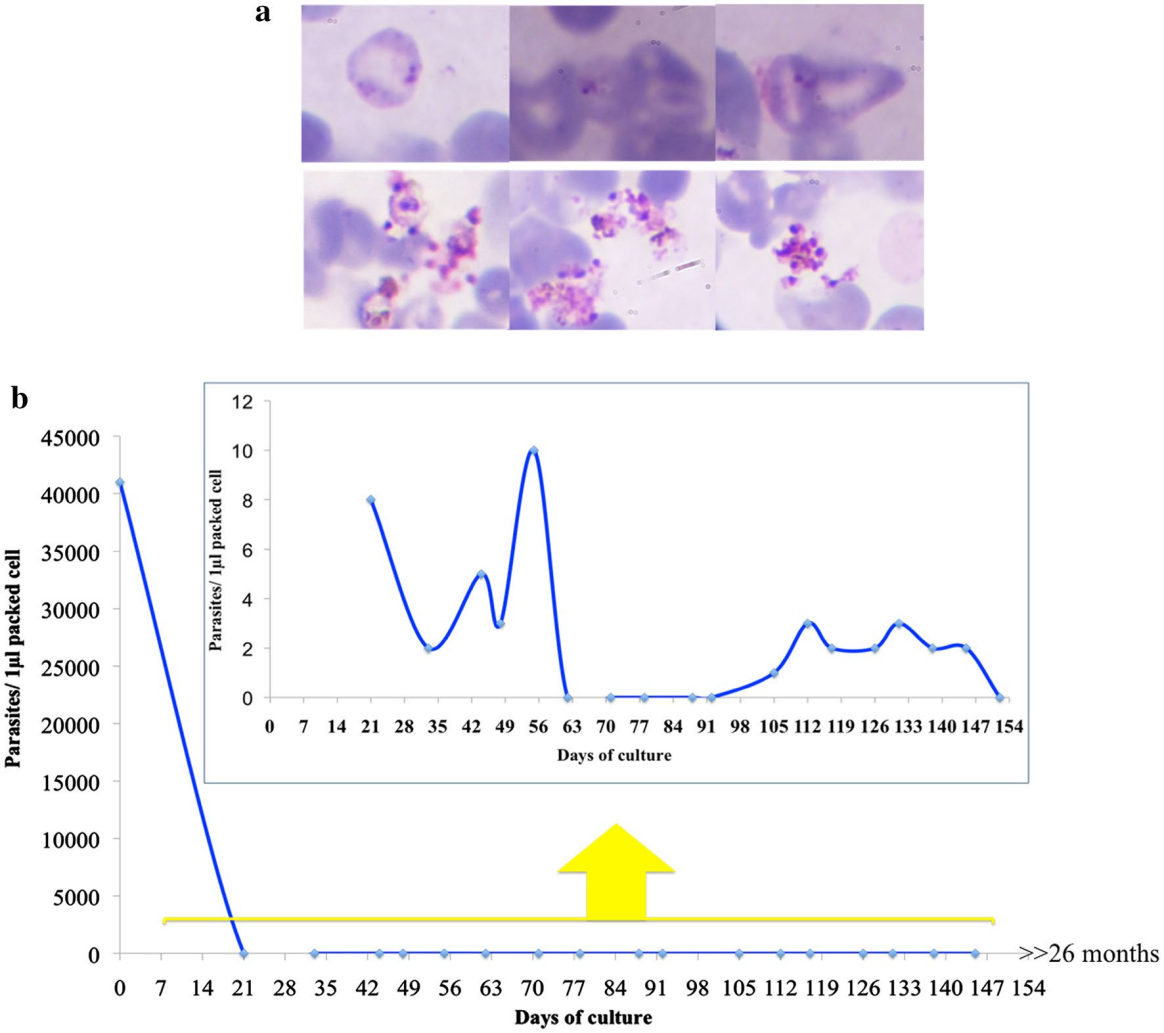
Giemsa-stained 1 month old-parasites and dynamic of parasitaemia of parasite cultured with MMM medium. **a** Giemsa stained-ring (*upper panel*) and ruptured schizont stage (*lower panel*) of the same parasite isolate cultured in MMM medium at 1 month. The present of ring stage parasites indicate the successful development and reinvasion of the parasites. **b** The fluctuation of parasitaemia of the parasites cultured in MMM medium. Parasite density was dramatically dropped during the first week of cultured and then maintained at very low density for the whole culture period.

Whereas the standard McCoy’s 5A medium has been widely used for *P. vivax* culture, delayed maturation of parasites in the second cycle after reinvasion of reticulocytes was observed, which reflected a delayed death type of phenotype. In most cases there was a noticeable lengthening of the blood-stage asexual development cycle, since the parasites were not able to complete schizogony within 48 h. To determine whether the inclusion of additional nutrients could improve parasite growth during in vitro culture, McCoy’s 5A complete medium was supplemented with ascorbic acid 0.0116 g/L (ASC), cholesterol 750 mg/L (CHO), reduced glutathione 2.2 mM (GSH), hypoxanthine 360 µM (HT), orotic acid 360 µM (ORA), inosine 5′-monophosphate 360 µM (IMP) and d-glucose 4 g/L (GLU). Each additive was evaluated individually and then in combination for the ability to improve parasite growth over a 7-day period in culture, although there was not a significant difference in parasitaemia among the media tested (Fig. 5). In contrast, a combination of all additives appeared to promote in vitro growth of the parasite, since only *P. vivax* parasites cultured in the Modified McCoy’s 5A medium (MMM) could develop to late schizont stage within 48 h (Fig. 6). Moreover no parasites persisted for a month in cultures with McCoy’s 5A complete medium containing no or single nutrient additives, but parasites were still detected in cultures with MMM (2–10 parasites/1 µL packed cells) (Fig. 7a). Therefore, MMM was selected as the medium for long-term culture of *P. vivax* parasite.

### Long-term culture of clinical isolates of *P. vivax*

Thirty *P. vivax* isolates were further tested in MMM in 24-well plates (500 µL/well) at 5% haematocrit with daily change of medium with RP added daily to maintain 4% reticulocytes. The parasites were cultured in ≥2 wells at the beginning for each isolates and parasite growth was monitored by Giemsa-stained thick and thin smears (1 µL packed cells) once a week. Parasite cultures were split once the haematocrit was higher than 5%. Most isolates shared a similar growth pattern with fluctuations of parasitaemia (Fig. 7b).

When enough cultured packed cell volume was available, 100 µL of packed cells from the cultures were used for parasite detection by nested-PCR. Considering the growth pattern of the parasite cultured mentioned above in this system, the culture wells that were negative by nested-PCR for four consecutive times were discarded. The RT-QMAL was also performed for all of the cultures. Three out of 30 isolates were positive by this method (see Additional file 3). The longest culture period was over 26 months.

### Screening of *P. vivax* isolates producing infective gametocytes

**Fig. 8:**
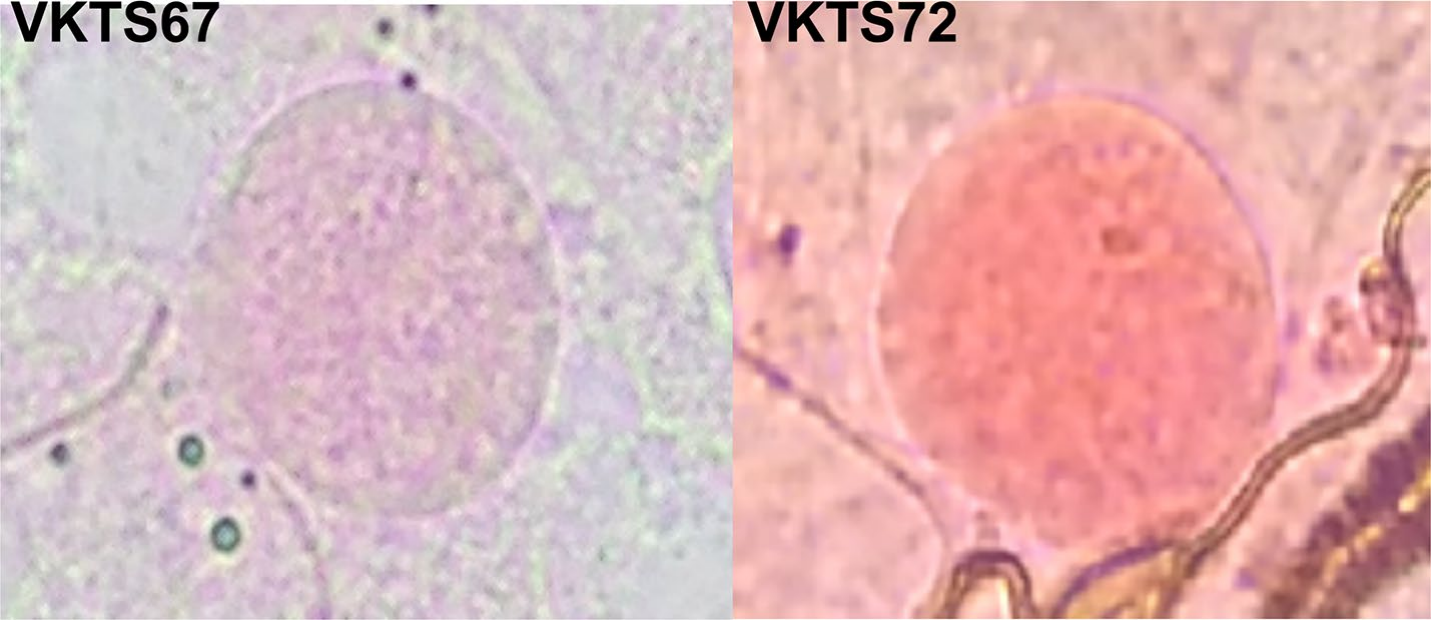
Oocysts on mosquito midguts stained with mercurochrome. Parasite cultures from 30 isolates were fed to female *Anopheles dirus* mosquitoes on day 7 of parasite culture using membrane feeding. Two out of 30 isolates were infective to mosquitoes with oocysts.

In order to determine whether gametocytes could form and mature using the MMM culture medium and protocol, aliquots of day 7 cultures were fed to *An. dirus* mosquitoes. Assuming a 3-day longevity of *P. vivax* gametocytes [14], the 7-day culture period was selected to make sure that the available parasites were not too low and gametocytes present in the culture were not the ones present in the original samples. After mosquito feeding, two out of 30 isolates were infective to mosquitoes (Fig. 8).

## Discussion

During the past decades, many efforts have been undertaken to establish a continuous culture system for *P. vivax.* Several established systems were shown to be able to support short-term growth with some potentially supporting longer-term maintenance of the parasites [2–7]. One of the major challenges in the development of in vitro culture of *P. vivax* lies in its preference to invade reticulocytes [15]. Provision of sufficient reticulocytes is a critical factor for the success of continuous culture of this parasite. In addition, *P. vivax* also invades Duffy positive (Fy antigen) reticulocytes [16, 17]. While testing Fy antigen is not performed by most of the blood banks, in-house testing could be done by using a simple agglutination test with commercially available reagents (ortho-clinical diagnostic) or flow cytometry using Fy^6^ antibody [18].

The level of available reticulocytes in *P. vivax* in vitro culture should be of particular importance since too many reticulocytes may dilute the parasite in culture, whereas too few may limit host cells for parasites to invade. In this study, the optimum level of the reticulocytes for parasite invasion was ~4%, whereas further increases did not result in any significant increase in parasitaemia. Various studies have used different sources of reticulocytes, including those from peripheral blood [5], haemochromatosis blood [6], and cord blood [4, 19]. The advantage of using reticulocytes from haemochromatosis blood is that they contain adult haemoglobin and the starting proportion of reticulocytes is high. However, since haemochromatosis occurs mainly in Caucasians, this type of reticulocytes for *P. vivax* culture is normally not accessible to researchers outside Europe or Americas. Another source of reticulocytes is cord blood, which contains a high percentage of young reticulocytes. Although the purified reticulocytes from cord blood can be cryopreserved in Glycerolyte 57 for later uses [19], they contain high-level fetal haemoglobin that could affect parasite development. A major breakthrough is the use of haematopoietic stem cell culture technology to generate highly pure young reticulocytes for *P. vivax* culture [7, 20, 21]. Yet, major limitations lie in the low reticulocyte yield and high cost of this method. Comparison of reticulocytes obtained from three different sources did not show significant differences in their abilities to support parasite invasion and maturation. Nevertheless, parasites grown in reticulocytes from peripheral blood seemed to develop better than those grown in reticulocytes from the other two sources.

A variety of reticulocyte purification procedures have been used to enrich reticulocytes for adding to *P. vivax* cultures. Differential centrifugation in autonomous plasma could purify reticulocytes for up to 20% from haemochromatosis blood and supported parasite growth without requiring additional reagents [6]. However, the method is labour-intensive and needs an ultracentrifuge. Several density gradient centrifugation methods have also been developed. The Percoll/Renografin-60 method could enrich reticulocytes up to 15% from adult peripheral blood, but Renografin-60 has toxic effect on the parasite [5]. Without Renografin-60, 71% Percoll centrifugation also allowed enrichment of reticulocytes from cord blood without toxicity to the parasite [9, 19], but this method seems less effective when using peripheral blood. A recently developed protocol for reticulocyte enrichment by using aqueous multiphase systems (AMPS) could obtain highly pure reticulocyte, but the yield was very low [22].

**Fig. 9:**
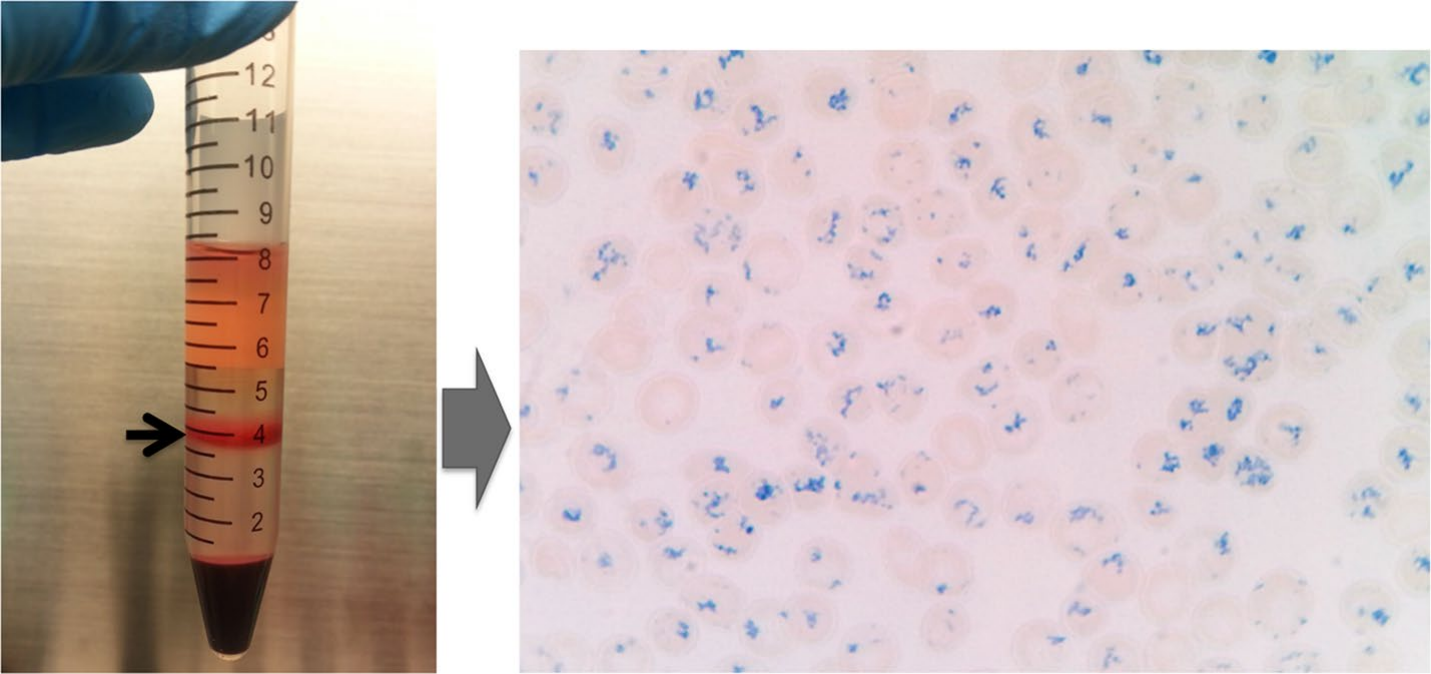
Purified reticulocytes stained with New Methylene Blue. Reticulocytes from peripheral blood were purified by 19% Nycodenz-KCl. After centrifugation at 3,000×*g* for 30 min, the reticulocyte-enriched fraction (*black arrow*, *left panel*) was collected from the interface and washed three times with RIM. The purified reticulocytes were stained with New Methylene Blue for 15 min before preparing the thin smears. The reticulocytes were identified as the *blue* reticulin-containing cells (*right panel*).

In this study, a simple and reproducible method for purifying reticulocytes from adult blood using 19% Nycodenz centrifugation was employed. Using this method, reticulocytes up to ~80% reticulocytes could be highly enriched from adult peripheral blood (with a normal range of 0.5–2% reticulocytes) to yield 0.5–2 mL packed cells from 30 mL of packed blood cells (Fig. 9). With this method, it requires only 1–2 reticulocyte purifications per week to maintain *P. vivax* cultures from 30 isolates with at least two wells per isolate. More importantly, reticulocytes purified by this method could support parasite invasion in vitro and in vivo [23] without any noticeable toxic effect on parasites. This method alleviates the difficulty in preparing reticulocytes during long-term *P. vivax* culture.

Regardless of the source of reticulocytes used for in vitro culture, delayed parasite maturation was observed. To determine whether this delayed parasite maturation was due to the lack of certain nutrients needed for parasite growth, different culture media and specific nutrient additives were evaluated to improve parasite growth. A combination of additives to McCoy’s 5A medium (MMM) was determined to support improved parasite growth and efficiency of schizont maturation better than other media during long-term culture.

## Conclusions

In summary, this study compared the effects of different culture variables, both of host origin and culture environments, in order to identify the optimum conditions for long-term culture of *P. vivax*. This is a further improvement of conditions for long-term culture of *P. vivax*. By using this system, three of 30 isolates could be maintained in vitro for with the longest culture period over 26 months, albeit parasite density was low. Future efforts should be directed towards increasing parasite density in culture in order to provide sufficient materials for studying parasite biology.

## Additional files

Aditional file 1. Parasite density of *P. vivax* cultured with different sources of reticulocytes in 7 days. Reticulocytes purified from peripheral blood (red line), cord blood (blue line) and hematopoietic stem cell (green line) were used to cultured fresh isolates of *P. vivax* (11 isolates). Reticulocytes were added to the cultures daily at a final 4% and the parasite density was determined for 7 days from Giemsa-stained thick smears (1 µl packed cells). Each line represents average parasite density from 7 days obtained from 11 *P. vivax *isolates. Aditional file 2. Gametocyte density of *P. vivax* cultured with different sources of reticulocytes in 7 days. Reticulocytes purified from peripheral blood (red line), cord blood (blue line) and hematopoietic stem cell (green line) were used to cultured fresh isolates of *P. vivax* (11 isolates). Reticulocytes were added to the cultures daily at a final 4% and the gametocyte density was determined for 7 days from Giemsa-stained thick smears (1 µl packed cells). Each line represents average parasite density from 7 days obtained from 11 *P. vivax* isolates. Cord blood-reticulocytes has highly expression of fetal hemoglobin but still shown better support gametocyte production in some parasite isolates (VKTS33, VKBT59, VKBT63, and VKBT81). Aditional file 3. The RT-QMAL result of *P. vivax *from in vitro cultures. The RT-QMAL was performed from 50 µl packed cells of parasite cultures. The standard curve was generated from assay-specific control plasmid at concentration of 102-106 copies/reaction. The total RNA of *P. falciparum* was used as a positive control. Three out of thirty isolates were positive for RT-QMAL, VKTS38, VKP7, and VKBT139
